# Simulational study of a dosimetric comparison between a Gamma Knife treatment plan and an intensity-modulated radiotherapy plan for skull base tumors

**DOI:** 10.1093/jrr/rrt136

**Published:** 2013-12-17

**Authors:** Hisato Nakazawa, Yoshimasa Mori, Masataka Komori, Takahiko Tsugawa, Yuta Shibamoto, Tatsuya Kobayashi, Chisa Hashizume, Yukio Uchiyama, Masahiro Hagiwara

**Affiliations:** 1 Department of Radiological Sciences, Nagoya University Graduate School of Medicine, Nagoya, Aichi, Japan; 2 Nagoya Radiosurgery Center, Nagoya Kyoritsu Hospital, Nagoya, Aichi, Japan; 3 Department of Radiology, Nagoya City University Graduate School of Medical Sciences, Nagoya, Aichi, Japan

**Keywords:** stereotactic radiosurgery, Gamma Knife, Perfexion, Novalis, intensity-modulated radiation therapy, intracranial lesions

## Abstract

Fractionated stereotactic radiotherapy (SRT) is performed with a linear accelerator-based system such as Novalis. Recently, Gamma Knife Perfexion (PFX) featured the Extend system with relocatable fixation devices available for SRT. In this study, the dosimetric results of these two modalities were compared from the viewpoint of conformity, heterogeneity and gradient in target covering. A total of 14 patients with skull base tumors were treated with Novalis intensity-modulated (IM)-SRT. Treatment was planned on an iPlan workstation. Five- to seven-beam IM-SRT was performed in 14–18 fractions with a fraction dose of 2.5 or 3 Gy. With these patients' data, additional treatment planning was simulated using a GammaPlan workstation for PFX-SRT. Reference CT images with planning structure contour sets on iPlan, including the planning target volume (PTV, 1.1–102.2 ml) and organs at risk, were exported to GammaPlan in DICOM-RT format. Dosimetric results for Novalis IM-SRT and PFX-SRT were evaluated in the same prescription doses. The isocenter number of PFX was between 12 and 50 at the isodose contour of 50–60%. The PTV coverage was 95–99% for Novalis and 94–98% for PFX. The conformity index (CI) was 1.11–1.61 and 1.04–1.15, the homogeneity index (HI) was 1.1–3.62 and 2.3–3.25, and the gradient index (GI) was 3.72–7.97 and 2.54–3.39 for Novalis and PFX, respectively. PTV coverage by Novalis and PFX was almost equivalent. PFX was superior in CI and GI, and Novalis was better in HI. Better conformality would be achieved by PFX, when the homogeneity inside tumors is less important.

## INTRODUCTION

Stereotactic radiosurgery (SRS) and fractionated stereotactic radiotherapy (SRT) using a Gamma Knife (GK) (Elekta, Tokyo) or a linear accelerator (LINAC) such as Novalis (BrainLAB, Tokyo) have been reported to be safe and effective treatment options for various brain disorders, including brain tumors [1–3]. Fractionated SRT has a radiobiological advantage for the protection of surrounding normal structures, especially when the tumor is large or involves critical structures such as cranial nerves in the skull base [4]. Novalis is equipped with a sophisticated patient positioning system, ExacTrac (BrainLAB) to perform SRT [5]. Meanwhile, the latest version of GK, Perfexion (PFX) (Elekta, Tokyo) makes the Extend system available for fractionated and multisession GK PFX treatment without using an invasive skull frame. These two modalities, Novalis and Gamma Knife, employ rather different radiation delivery systems, i.e. LINAC X-ray IMRT with multileaf collimators and a multi-isocenter with cone-collimated, multiresource gamma-ray beams, respectively. In this study, we made a dosimetric comparison of these two modalities using indices of conformity, heterogeneity and gradient in skull base tumors of various sizes involving or adjacent to optic pathways.

## MATERIALS AND METHODS

We used the image data of 14 patients with brain tumors previously treated by Novalis SRT. The Research Ethics Board of Nagoya University Graduate School of Medicine approved this clinical study (Approval No. 12-303). Novalis SRT plans were made on the iPlan (BrainLAB) workstation and were used for actual patient treatment. For this study, GK PFX-SRT plans for each patient were additionally made for simulation. The same CT image data and structure sets, including the planning target volume (PTV) and organs at risk (OARs) that were used by Novalis treatment, were transferred to the Leksell GammaPlan (LGP) version 10.1.1 treatment-planning workstation (Elekta, Tokyo) via a DICOM-RT (digital communications in medicine-radiation therapy) protocol from the iPlan treatment-planning workstation. The same MRI data were also transferred and were fused with the CT images. Multi-isocenter GK PFX plans were made. In this way, two plans, a real treatment Novalis SRT plan and a simulation GK PFX plan, were compared.

### Patients

We treated 14 patients with a skull base (*n* = 5) or cavernous sinus (*n* = 4) meningioma, craniopharyngioma (*n* = 4), or pituitary adenoma (*n* = 1) by intensity-modulated stereotactic radiotherapy (IM-SRT) using Novalis from April 2011 through June 2012. In this study, a GK PFX treatment plan was additionally made as a simulation plan for each patient. Actual treatment plans using Novalis and simulation plans of GK PFX were compared.

### Imaging protocol

The treatment-planning images were acquired with magnetic resonance imaging (MRI) using a 1.5-Tesla or 3.0-Tesla scanner (Signa Echo Speed Plus 1.5 T, Signa HDxt 3.0 T; GE Healthcare, Tokyo) and 4-detector computed tomography (CT) (Light Speed Plus; GE Healthcare, Tokyo). The references for dose calculation in treatment planning were the CT images. A CT image resolution of 512 × 512 pixels in the axial plane and slice thickness of 1.25 mm was adopted to reduce partial volume effects. To determine gross tumor volume (GTV), contrast-enhanced CT and MRI were acquired. Conditions for non-contrast and contrast-enhanced CT were the same except for the size of the field of view. The slice thickness of MRI was specified from 1–2 mm depending on the tumor size by 3D-SPGR (3D fast-spoiled gradient-recalled acquisition in the steady state) sequence with gadolinium enhancement and 3D fast spin echo.

### Novalis treatment planning

All 14 patients underwent treatment-planning CT and MRI. Planning CT and MRI were fused on the iPlan RT Image version 4.1.2 (BrainLAB). Delineation of target and risk organs was performed with an autosegmentation function and manually by a radiation oncologist and a neurosurgeon on iPlan RT image treatment-planning software. The PTV margin was determined to be 2 mm, considering the spatial uncertainty, including the patient setup error, isocenter mechanical deviation, and ExacTrac image-guidance error [6]. When the CTV was in close contact with critical organs, the CTV-PTV margin was adjusted manually to avoid overlapping the PTV and these critical organs. The Novalis equipped with a micro-multileaf collimator (mMLC) with 3-mm thick leaves (m3; BrainLAB) was used. SRS/SRT with the Novalis system has been described previously [2, 3, 5]. The targets were covered with a ≥ 95% isodose level. The PTV ranged from 1.1–102.2 ml (median, 19.5 ml). The algorithm of dose calculation in iPlan RT dose version 4.1.2 software was the pencil beam convolution (PBC) method. Dosing for all patients was planned with a single isocenter IMRT by radiation oncologists and neurosurgeons. Parameter evaluation of the dose–volume histogram (DVH) was performed considering target coverage and the dose limitation for OARs. The dose constraints for OARs used in the IMRT optimization process were determined according to tolerance dose; e.g. 55 Gy for brain stem (0.1 ml-volume), 50 Gy for optic nerve (0.1 ml), 10 Gy for lens (max), 50 Gy for eye (1 ml), and 50 Gy for acoustic nerve (0.1 ml) with a 2 Gy per fraction regime. The patients underwent IM-SRT calculated by PBC with 6-MV photon beams in 14–18 fractions to a total dose of 40–48 Gy (median 42.5 Gy) (at 100% isodose = at normalization point) over 3–4 weeks. The IM-SRT treatment times were estimated for a dose rate of 320 monitor units/min, calculated in log files of patient management software for treatment.

### GK planning

The same CT image data and structure sets including PTV and OARs of patients that were used in Novalis treatment were transferred to LGP version 10.1.1 treatment-planning software from iPlan treatment-planning software via a DICOM-RT protocol. We determined a 2-mm PTV margin around the GTV (= CTV, clinical target volume) in multisession GK-SRT using the Extend system. The dose algorithm, available in LGP software is a simple tissue maximum ratio (TMR) 10 method employing the measurement-based dose calculation by replacing all anatomical structures with water-equivalent material [7]. Multi-isocenter beam delivery, different from the Novalis single isocenter technique, was used to cover the target volume. The OARs were spared as much as possible (e.g. optic nerves less than approximately half of the prescribed isodose) even when it was adjacent to the target. The prescribed dose-fractionation schedule for the target was defined as the same as that used in Novalis IM-SRT. The treatment times with GK PFX-SRT were also estimated using a dose rate of 2.722 Gy/min for ^60^Cobalt, calculated by LGP treatment software.

### Dosimetric analysis

In this study, we evaluated each dosimetric characteristic using dosimetric metrics of both Novalis IM-SRT as LINAC-based SRT and multisession GK PFX-SRT using the Extend system with a relocatable frame system. We used the dose-calculation algorithm of PBC for the Novalis plan and TMR 10 for the GK plan.

PFX-SRT and IM-SRT multibeam treatment planning was compared to characterize each irradiated technique using dosimetric metrics. These include the PTV coverage (ratio of the target volume within the prescribed isodose), conformity index (CI, ratio of the prescription volume to the target volume) according to the RTOG (Radiation Therapy Oncology Group) radiosurgery guidelines, 1993 [8], gradient index (GI) reflecting the degree of steepness of dose fall-off outside the target volume, defined as the ratio of the volume of half the prescription isodose to the prescription isodose volume, as described by Paddick *et al*. [9], and radical dose homogeneity index (rHI) introduced by minimum and maximum doses within the target to evaluate the feature for the target of the treatment plans [10]. In addition, for each patient, the irradiation treatment times, excluding patient setup and treatment interruption as a result of bathroom breaks, were calculated.

### Statistical analysis

Collected dosimetry data were analyzed using SPSS version 18.0 (IBM, Japan). The paired t test was used to examine differences between indices of Novalis IM-SRT and those of PFX-SRT treatment plans. Differences with *P* < 0.05 were regarded as significant.

## RESULTS

**Fig. 1.**
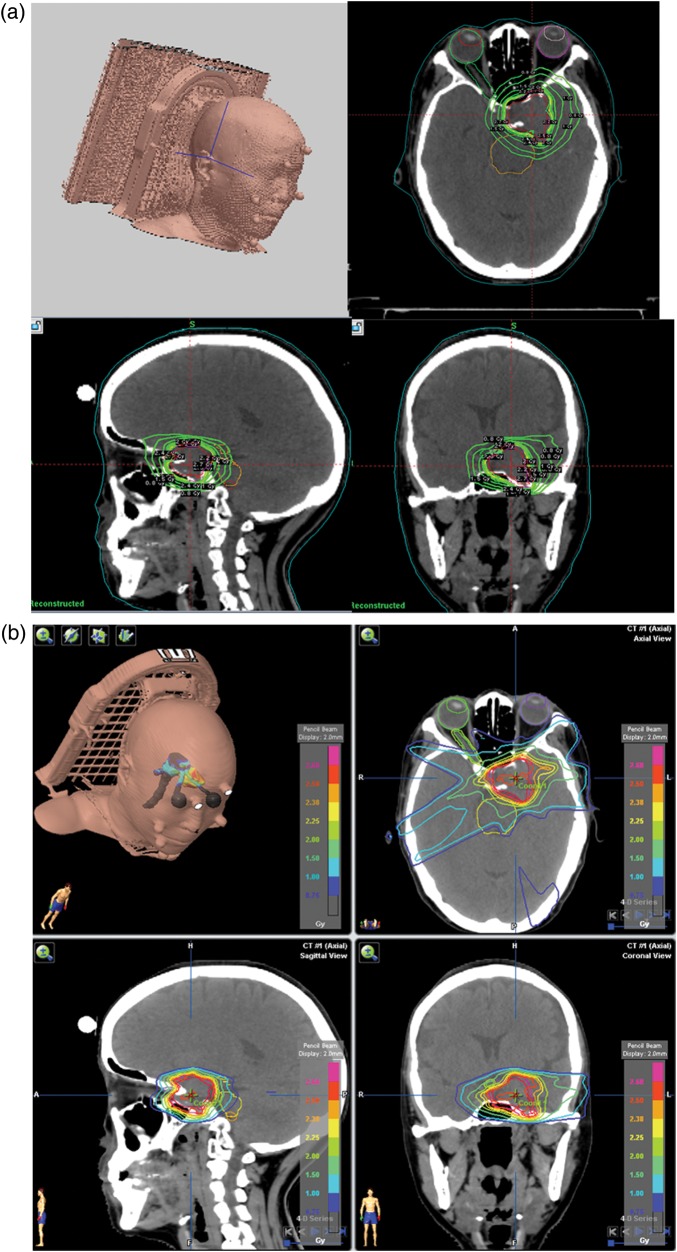

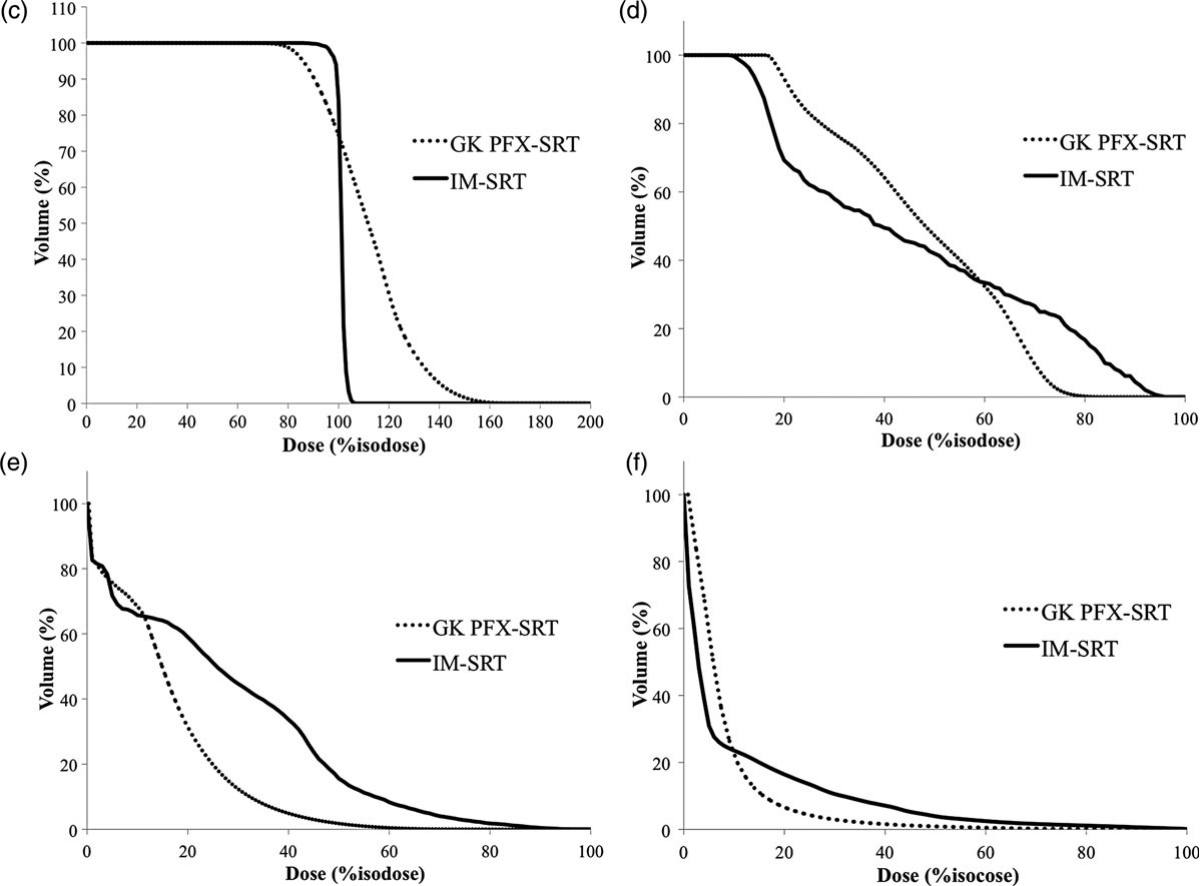
Dose distributions and dose–volume histograms (DVHs) in a patient with a small skull base tumor (patient A). Axial (right upper), coronal (right lower), and sagittal (left lower) views of the multi-isocenter Gamma Knife (GK) Perfexion (PFX)-stereotactic radiotherapy (SRT) plan (**a**). Axial (right upper), coronal (right lower), and sagittal (left lower) views of coplanar 5-beam intensity-modulated SRT (**b**). DVHs for the PTV (**c**), ipsilateral optic nerve (**d**), brain stem (**e**) and normal brain tissue (**f**). GK PFX-SRT provided lower percentage isodose for PTV coverage. GK PFX-SRT tended to deliver lower doses to the OARs than IM-SRT.

**Fig. 2.**
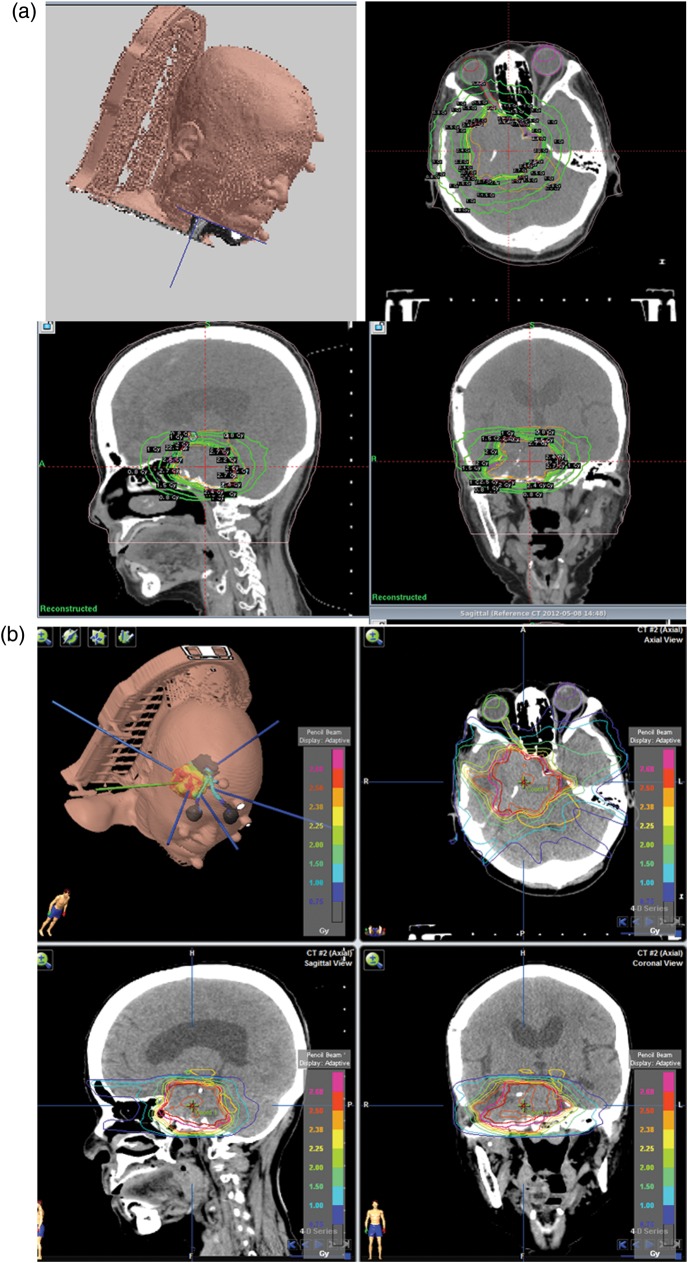

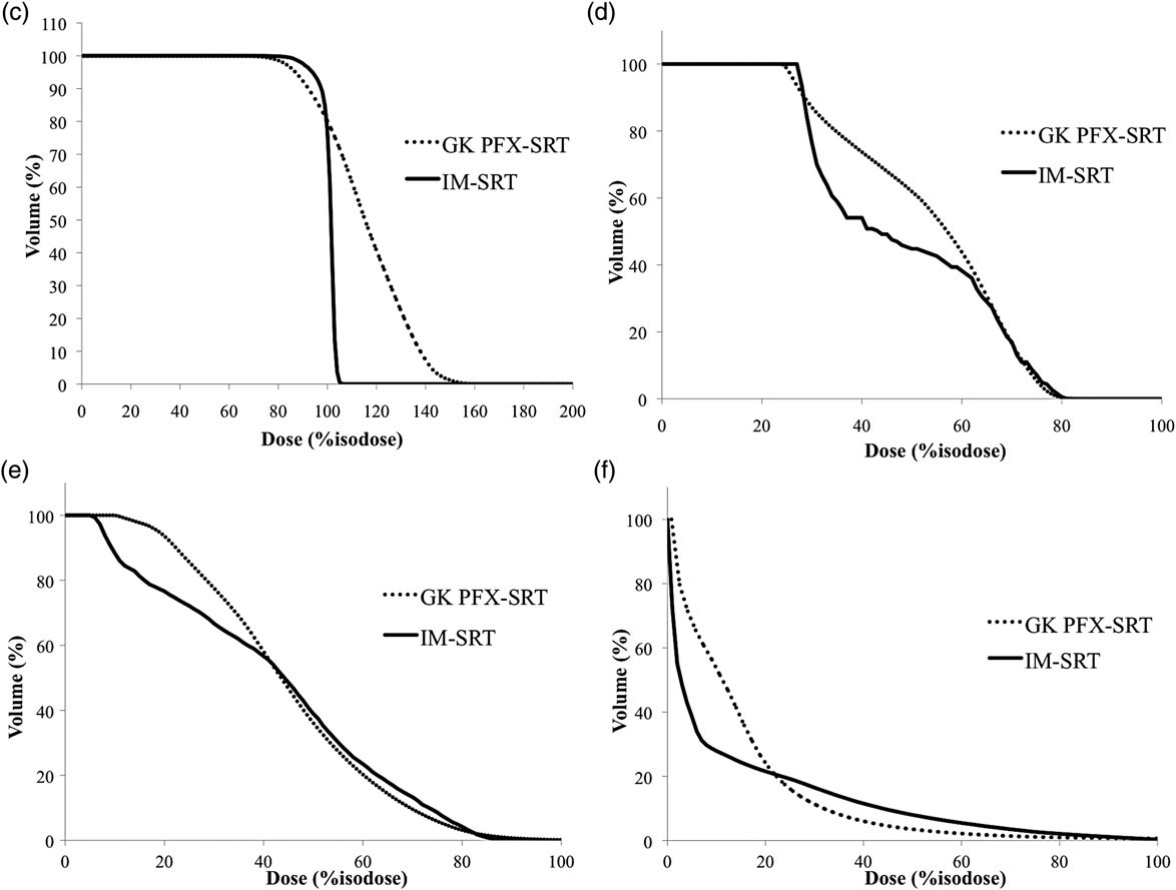
Dose distributions and dose–volume histograms (DVHs) in a patient with a large skull base tumor (patient H). Axial (right upper), coronal (right lower), and sagittal (left lower) views of the multi-isocenter Gamma Knife (GK) Perfexion (PFX)-stereotactic radiotherapy (SRT) plan (**a**). Axial (right upper), coronal (right lower), and sagittal (left lower) views of coplanar 5-beam intensity-modulated SRT (**b**). DVHs for the PTV (**c**), ipsilateral optic nerve (**d**), brain stem (**e**) and normal brain tissue (**f**). GK PFX-SRT provided lower percentage isodose for PTV coverage. GK PFX-SRT and IM-SRT were almost equivalent in dose distribution in the OARs.

The treatment parameters of 14 patients created with both PFX-SRT and Novalis IM-SRT are shown in Table 1. The PTV volume ranged from 1.1–102.2 cc (median 19.45) and the isocenter number of PFX-SRT was between 12 and 50 (median, 36) to the 50–60% isodose contour (mean 50). The PTV coverage ranged from 94–98% (mean, 97%) for PFX and from 95–99% (96.6%) of D95 (delivery dose for 95% volume of PTV) for Novalis. The estimated irradiation time ranged from 12–39 min for PFX and 8–11 min for Novalis. There was a significant difference between the mean beam-on time for PFX and Novalis (24 vs 8 min.; *P* < 0.001). The dosimetric parameters of the PTV are presented in Table 2. The CI was 1.04–1.15 and 1.11–1.61 with PFX and Novalis, respectively. The mean CI was significantly smaller for PFX than for Novalis (1.09 vs 1.36; *P* < 0.001). The HI was 2.3–3.25 and 1.1–3.62 with PFX and Novalis, respectively. The mean HI was significantly larger for PFX than for Novalis (2.75 vs 1.61; *P* < 0.001). The GI was 2.54–3.39 and 3.72–7.97 with PFX and Novalis, respectively. The mean GI was significantly smaller for PFX than for Novalis (2.84 vs 5.53; *P* < 0.001). Figures 1 and 2 show dose distributions and dose–volume histograms of representative cases planned using both PFX-SRT and Novalis IM-SRT. Dose distributions in OARs (the optic apparatus, brain stem and normal brain tissue) in PFX-SRT were almost equivalent to or better than those in IM-SRT.

**Table 1.** Treatment parameters of GK PFX-SRT and Novalis IM-SRT

Case number	PTV (cc)	IMRT technique	PFX isocenter number	IMRT D95 isodose (%)	PFX coverage/pr. isodose (%)	IMRT treatment time (min)	PFX treatment time (min)
A	19.7	5 field CP	31	99	98/50	10	20
B	17.2	5 field CP	26	96	98/50	8	27
C	19.2	5 field CP	37	95	96/50	8	39
D	31.2	5 field CP	46	95	97/50	10	20
E	55.1	7 field CP	37	96	98/50	10	29
F	60.1	5 field CP	35	98	96/50	9	22
G	102.2	7 field CP	50	96	95/50	11	28
H	78.2	6 field CP	40	95	97/50	11	27
I	51.2	5 field CP	40	98	98/50	11	21
J	14.8	5 field CP	32	98	97/50	8	26
K	1.1	5 field CP	12	96	94/50	7	12
L	7.9	5 field CP	33	98	94/60	7	17
M	2.3	5 field CP	21	96	96/50	10	20
N	14.6	5 field CP	38	97	98/55	8	22
Mean	33.9		34.1	96.6	97/50	9	24

PTV = planning target volume, IM = intensity modulation, SRT = stereotactic radiotherapy, GK = Gamma Knife, PFX = Perfexion, CP = coplanar, pr. = prescribed.

**Table 2.** Dosimetric indices of GK PFX-SRT and Novalis IM-SRT

Case number	IMRT CI	PFX CI	IMRT rHI	PFX rHI	IMRT GI	PFX GI
A	1.25	1.11	1.34	3.25	6.03	2.58
B	1.48	1.14	1.8	2.63	5.63	2.63
C	1.61	1.12	1.54	3.13	6.78	3.39
D	1.59	1.07	1.58	2.47	6.58	2.76
E	1.38	1.09	1.9	2.86	4.42	2.8
F	1.28	1.07	1.6	2.54	3.72	3.02
G	1.5	1.07	3.62	2.78	4.61	3.05
H	1.35	1.07	1.53	2.94	4.53	2.54
I	1.28	1.08	1.74	2.65	4.32	2.7
J	1.11	1.11	1.17	2.68	4.04	2.8
K	1.36	1.04	1.14	2.94	7.97	3.09
L	1.12	1.07	1.1	2.53	4.94	2.9
M	1.48	1.12	1.23	2.83	7.93	2.86
N	1.3	1.15	1.28	2.3	5.95	2.61
Mean	1.36	1.09	1.61	2.75	5.53	2.84

CI = conformity index, rHI = radical dose homogeneity index, GI = gradient index, other abbreviations as in Table 1.

## DISCUSSION

PFX, the latest version of GK, has an improved automatically controlled collimator setting incorporating movable ^60^Cobalt sources and a stationary collimator configuration. The collimator consists of eight sectors providing different beam diameters of 4, 8 and 16 mm, as well as the beam block position [7]. In GK, SRS with rigid skull frame fixation was usually only performed with MRI, and we included no PTV margin in dose planning. We have already confirmed that the positional error between MRI and CT images was minimal, < 0.8 mm in our commissioning test [11]. On the other hand, in IM-SRT using a thermoplastic head shell, appropriate margins of 1–2 mm are generally added [3, 5, 6]. A 2-mm margin is necessary in planning for intracranial lesions in our facility on the evidence of our previous physical experiment [6]. In this study, we used the same PTV margin of 2 mm for both GK-PFX SRT and Novalis SRT. Several reports of clinical experiences with the Extend system for multisession SRS have already been published [12–14]. Ruschin *et al*. [13] reported a repositioning error of 1.3 mm at the 95% confidence limit. Schelesinger *et al*. [14] investigated inter- and intra-fractional patient positioning and immobilization in GK Extend, and suggested that a margin of 0.81 mm is required for 90% of patients to receive >95% of the planned dose. Considering these reports, a PTV margin of 2 mm is thought to be sufficient and reasonable.

This study compared GK PFX-SRT and Novalis IM-SRT in 14 skull base tumors of variable volumes. PTV coverage in GK PFX-SRT and D95 in Novalis IM-SRT was equivalent. However, CI and GI were superior in PFX, while HI was better in Novalis. These metrics did not correlate with the size of the PTV. Several papers have compared CI between GK and other modalities [15–17). In a phantom study, Kumar *et al*. [15] experimented with an anthropomorphic head phantom to make a dosimetric comparison of TomoTherapy versus GK model C in various size targets. They reported that conformity was equivalent between the two techniques. Ma *et al*. [16] reported that fan-beam IM-SRS plans were equivalent to GK model U plans. Nakamura *et al*. [17] compared single-session radiosurgery plans between GK model C SRS and Novalis IM-SRS in small- and medium-sized skull base tumors. It was reported that the CI of Novalis IM-SRS was superior to that of model C SRS, which seemed contrary to the results in our study, although they employed more complicated beam delivery in IM-SRS planning. In our study, the CI of GK PFX-SRT was higher than that of Novalis IM-SRT. We used larger targets, more than 3-fold in volume. The mean number of isocenters was 34.1 in the PFX SRT plan in our study, which was about three times as many as in their study (mean isocenter number, 13.2). In GK SRS or SRT, CI increases with isocenter numbers. The brand-new version of GK, PFX, is equipped with an automatic collimator arrangement system without requiring manual collimator exchange, and a couch-traveling system that is approximately 10 times faster than model C, so treatment time with many isocenters is considered to be in a clinically acceptable range. In our study, the same datasets of PTV and OARs were used to perform an accurate intercomparative study of dosimetric parameters between the two modalities. Recently, the DICOM-RT transfer function has become available between different treatment-planning systems.

This investigation is merely a modeling study. GK PFX-SRT plans of multifractions over at least 14 were made only for simulation. Clinically, hypofraction SRT with up to five fractions using the GK PFX Extend system has been performed [12–14]. We believe that SRT with more fractions in GK PFX and LINAC-based SRT have significant advantages in tumor control and for surrounding OARs, especially when optic pathways are adjacent to the tumors. Dose fractionation has been re-evaluated as definitely biologically important strategy in SRT for benign brain tumors [2]. Recently, PFX equipped with a cone-beam on-board imaging system has been developed [18], which should improve the accuracy of patient setup, with only a thermoplastic head shell, and is generally used in an LINAC-based system, which would make fractionated or multisession procedures easier.

PFX LGP and Novalis iPlan employ different dose-calculation algorithms, TMR10 and PBC. TMR10 is the water-based dose-calculation algorithm and does not consider tissue heterogeneity [7]. On the other hand, PBC performs only one-dimensional longitudinal correction for tissue inhomogeneity and does not consider lateral electron scattering. Recently Convolution and X-ray Voxel Monte Carlo (XVMC) run on each workstation. Both are expected to perform more accurate dose calculation. Comparison of these algorithms is to be investigated in the near future.

## CONCLUSION

In conclusion, our study showed that GK PFX-SRT is superior in conformality and gradient, while Novalis IM-SRT is superior in homogeneity. Further work is required to establish the clinical efficacy and physical validation of dose fractionation in GK PFX.

## CONFLICTS OF INTEREST

The authors report no conflicts of interest concerning the materials or methods used in this study or the findings specified in this paper.
